# Small Molecule Chaperones for the Treatment of Gaucher Disease and *GBA1*-Associated Parkinson Disease

**DOI:** 10.3389/fcell.2020.00271

**Published:** 2020-05-19

**Authors:** Tae-Un Han, Richard Sam, Ellen Sidransky

**Affiliations:** Medical Genetics Branch, National Human Genome Research Institute, National Institutes of Health, Bethesda, MD, United States

**Keywords:** gaucher disease, Parkinson disease, lysosome, glucocerebrosidase, *GBA1*, small molecule chaperones

## Abstract

Parkinson disease, the second most common movement disorder, is a complex neurodegenerative disorder hallmarked by the accumulation of alpha-synuclein, a neural-specific small protein associated with neuronal synapses. Mutations in the glucocerebrosidase gene (*GBA1)*, implicated in the rare, autosomal recessive lysosomal disorder Gaucher disease, are the most common known genetic risk factor for Parkinson disease. Insights into the inverse relationship between glucocerebrosidase and alpha-synuclein have led to new therapeutic approaches for the treatment of Gaucher disease and *GBA1-*associated Parkinson disease. Unlike the current drugs used to treat Gaucher disease, which are highly expensive and do not cross the blood-brain-barrier, new small molecules therapies, including competitive and non-competitive chaperones that enhance glucocerebrosidase levels are being developed to overcome these limitations. Some of these include iminosugars, ambroxol, other competitive glucocerebrosidase inhibitors, and non-inhibitory chaperones or activators that do not compete for the active site. These drugs, which have been shown in different disease models to increase glucocerebrosidase activity, could have potential as a therapy for Gaucher disease and *GBA1-* associated Parkinson disease. Some have been demonstrated to reduce α-synuclein levels in pre-clinical studies using cell-based or animal models of *GBA1*-associated Parkinson disease, and may also have utility for idiopathic Parkinson disease.

## Introduction

Gaucher disease (GD), first described in 1882 by Dr. Phillipe Gaucher, is an inborn error of metabolism due to mutations in the gene *GBA1*, encoding the lysosomal enzyme glucocerebrosidase (GCase) (Sidransky, 2012). GCase, a lysosomal hydrolase, cleaves both glucosylceramide (GlcCer) to ceramide and glucose, and glucosylsphingosine (GlcSph) into sphingosine and glucose. This rare autosomal recessive inherited disorder occurs in approximately 1:40,000–60,000 live births in the general population, whereas among Ashkenazi Jews the frequency increases to around 1:850 (Horowitz et al., 1998). In patients with GD, “Gaucher cells,” macrophages engorged with lysosomal GlcCer, present in the spleen, liver, and bone marrow, lead to organomegaly and inflammation (Pastores et al., 2000; Beutler and Grabowski, 2001; Sidransky, 2012). Clinically, there are three types of GD. Type 1 or non-neuronopathic GD presents with organomegaly, bone manifestations, anemia, and thrombocytopenia (Pastores et al., 2000; Beutler and Grabowski, 2001). Acute neuronopathic GD2, the rarest form with devastating and progressive neurodegeneration including bulbar findings, seizures, and opisthotonos, results in death during infancy or early childhood (Beutler and Grabowski, 2001; Sidransky, 2004; Weiss et al., 2015). Chronic neuronopathic GD3 is characterized by a slower progression of neurological symptoms, with a later onset. These patients have extensive visceral and skeletal involvement, but also may exhibit myoclonic epilepsy, ataxia, impaired eye movements, developmental delay, or intellectual deterioration (Sidransky, 2004, 2012; Gupta et al., 2011; Sidransky and Lopez, 2012). There is considerable phenotypic variation within each type of GD, including rare patients who develop Parkinson disease (PD).

Parkinson disease, first described in 1817 by James Parkinson, is found in 2–3% of individuals over age 65 (Poewe et al., 2017). This disease is characterized by the loss of dopaminergic (DA) neurons in the substantia nigra, as well as by Lewy bodies containing alpha-synuclein (α-Syn) (Spillantini et al., 1997; Surmeier et al., 2017), which ultimately result in the classically associated symptoms, tremor, postural instability, bradykinesia, and rigidity (Poewe et al., 2017). Currently, mutations in *GBA1* are one of the most common genetic risk factors for PD, as well as dementia with Lewy bodies (DLB). Mutations in *GBA1* were found in 5–20% of sporadic PD cases with an estimated odds ratio of 5.4. In DLB the odds ratio was 8.3 (Sidransky et al., 2009; Nalls et al., 2013; Mullin et al., 2019). Patients with *GBA1-*associated PD (*GBA1*-PD) resemble those with sporadic PD but exhibit an earlier disease onset. The lysosome, the central player uniting GD and PD, is the main organelle responsible for α-Syn degradation (Sala et al., 2016). While the mechanism underlying the association between GD and PD still remains controversial, one hypothesis proposed posits that elevated substrate levels resulting from diminished GCase could stabilize soluble oligomeric α-Syn intermediates, enhancing oligomerization and accumulation of α-Syn within lysosomes (Yap et al., 2011; Taguchi et al., 2017). Increased levels of α-Syn in DA neurons could also inhibit the translocation of GCase from the endoplasmic reticulum (ER) to lysosomes which could lead to a gradual increase of the lipid substrates within the lysosome (Mazzulli et al., 2016a). This positive feedback loop with the combination of inhibited GCase trafficking and α-Syn accumulation may result in neurodegeneration (Balestrino and Schapira, 2018; Stojkovska et al., 2018). Furthermore, even patients with PD without *GBA1* mutations have lower levels of GCase, suggesting that in idiopathic PD, reduced GCase activity may also contribute to disease progression (Gegg et al., 2012; Murphy et al., 2014).

Due to this apparent reciprocal association between GCase and α-Syn, attention is being directed toward designing therapeutics for GD with implications for PD. Currently, patients with type 1 GD are commonly treated with enzyme replacement therapy (ERT) (Jung et al., 2016) which alleviates the hematological, visceral, and sometimes skeletal manifestations, but does not cross the blood-brain-barrier, and thus does not impact neurological involvement or PD manifestations (Valayannopoulos, 2013). Substrate reduction therapy (SRT), a second approach for treating GD, focuses on upstream targets, ultimately inhibiting the accumulation of GlcCer (Lukina et al., 2019). Two SRT drugs approved by FDA have shown efficacy in type 1 GD (Schiffmann et al., 2008; Bennett and Turcotte, 2015) and some cross the blood-brain-barrier. However, the current SRTs have not been efficacious for neuronopathic GD (nGD) (Bennett and Turcotte, 2015; Mistry et al., 2018; Zimran et al., 2018) and there are several pharmacokinetic limitations (Henley et al., 2014).

Pharmacological chaperones, small molecules designed to bind to a specific target protein and assist in the folding of the protein, are also being developed as an alternative treatment approach for GD or GD-associated PD. They further function by stabilizing the enzyme to prevent misfolding and to enhance accurate translocation of the protein from the ER to the Golgi and lysosome (Compain et al., 2006; Lieberman et al., 2007). Since most mutations encountered in GD or *GBA1*-PD are missense mutations leading to misfolded enzyme, pharmacological chaperones which cross the blood-brain-barrier provide an attractive therapeutic strategy. Here we review studies of chaperones as a treatment for GD and *GBA1*-PD, focusing on preclinical developments.

## Iminosugars

The first chaperone considered for GCase was an iminosugar, *N*-(*n*-nonyl) deoxynojirimycin (NN-DNJ). Sawkar et al. (2002) reported that the addition of NN-DNJ to fibroblast culture medium increased the activity of GCase in N370S mutant and wild-type but not L444P mutant cells. However, NN-DNJ also inhibits α-glucosidase and GlcCer synthase, rendering it difficult to use as a target-specific drug. Another iminosugar, α-1-C-Nonyl-DIX that specifically inhibits GCase, but not α-glucosidase, doubled the residual cellular activity of GCase in N370S/N370S fibroblasts (Compain et al., 2006). Bicyclic nojirimycin (NJ) analogs with a sp2-iminosugar structure were found to be stronger chaperones as well as selective and competitive inhibitors of lysosomal GCase (Luan et al., 2009, 2010; Tiscornia et al., 2013). Some of the new sp2-iminosugar derivatives inhibited enzyme activity by 10-fold at pH 5 (Mena-Barragan et al., 2016).

Another type of iminosugar, isofagomine (IFG), was reported to bind to the GCase active site, increase GCase activity in cell lysates and restore lysosomal trafficking of GCase in N370S cells (Lieberman et al., 2007). Incubation of GD patient-derived lymphoblastoid or fibroblast lines with IFG increased GCase activity 3.5- and 1.3-fold and reduced endogenous GlcCer levels (Khanna et al., 2010). Testing the effects of IFG on GCase activity in a complex nGD mouse model, 4L;C^∗^ (V394L/V394L + saposin C−/−) showed that IFG administration extended lifespan and increased GCase activity and protein levels in the brain and visceral tissue, with attenuation of proinflammatory responses (Sun et al., 2011). Further, *in vivo* tests of IFG in homozygous V394L, D409H, and D409V mice, which are nGD models demonstrated increased GCase activity in visceral tissues and brain extracts (Sun et al., 2012). Whether the effect of IFG on wild-type GCase could be beneficial in synucleinopathies was evaluated using mice overexpressing human wild-type α-Syn (Thy1- α-Syn). Treating with IFG (AT2101) orally for 4 months improved motor function, diminished microglial inflammatory response in the substantia nigra, reduced α-Syn immunoreactivity in nigral DA neurons, and reduced small α-Syn aggregates (Richter et al., 2014). Another study in 4L;C^∗^ mice reported that IFG did not alter the GlcCer and GlcSph accumulation, but attenuated disease progression and altered global expression profiles of brain mRNA and miRNAs (Dasgupta et al., 2015). Treating *Drosophila* manipulated to express human wild-type, N370S and L444P *GBA1*, with IFG resulted in decreased ER stress and preserved motor function, suggesting that IFG might have potential as a PD therapy (Sanchez-Martinez et al., 2016).

De La Mata et al. reported that a distinct iminosugar, NAdBT-AIJ restored mitochondrial dysfunction and GCase activity in L444P mice in combination with coenzyme Q10 (De La Mata et al., 2015). Another pyrrolidine-based iminosugar, α-1-C-tridecyl-DAB (5j) was shown to function as a chaperone inhibitor of GCase enhancing enzyme activity at concentrations 10 times lower than IFG (Kato et al., 2016).

## Ambroxol

Ambroxol (ABX), a drug used to treat airway mucus hypersecretion and hyaline membrane disease in newborns, was found to be a pH-dependent, mixed-type inhibitor of GCase, and thus a potential therapy for GD. With its inhibitory activity maximal at neutral pH, ABX is found in the ER, and is undetectable at the acidic pH of lysosomes (Maegawa et al., 2009). GCase activity was enhanced in ABX-treated GD fibroblasts and lymphoblasts. Furthermore, modeling studies indicated that ABX interacts with both active site and non-active site residues of GCase (Maegawa et al., 2009). The tolerability and efficacy of ABX was evaluated in 12 patients with type 1 GD not receiving ERT. While only three patients who continued on ABX for a year had improved platelet counts and decreased organ volumes, the others remained stable, supporting the need for a larger clinical trial (Zimran et al., 2013). The chaperone activity and cytotoxicity of ABX was tested *in vitro*, demonstrating low cytotoxicity and significantly increased GCase activity in GD and GD+PD fibroblasts with different GD mutations, without any serious adverse effects (Bendikov Bar et al., 2013; Luan et al., 2013; McNeill et al., 2014). Another study in *GBA1*-mutant fibroblasts showed that ABX enhanced GCase activity by increasing Sap C and LIMP-2 protein levels (Ambrosi et al., 2015). Testing *in vivo* efficacy of 12 days of ABX in wild-type, L444P carrier and transgenic mice overexpressing human α-Syn demonstrated increased brain GCase activity and decreased total and phosphorylated α-Syn levels (Migdalska-Richards et al., 2016). In nonhuman primates, daily administration of ABX increased brain GCase activity, supporting clinical testing in humans (Migdalska-Richards et al., 2017a). In *Drosophila melanogaster* with a mutated *GBA1b* ortholog, treatment with ABX did not rescue GCase activity, but did ameliorate the unfolded protein response, inflammation and neuroinflammation, and enhance the life span (Cabasso et al., 2019). Several pilot clinical studies of ABX were performed in patients with nGD (Narita et al., 2016; Pawlinski et al., 2018; Kim et al., 2019). One study in five patients with GD3 showed that high-dose oral ABX had good safety and tolerability, significantly increased lymphocyte GCase activity, permeated the blood–brain barrier, and decreased GlcSph levels in the cerebrospinal fluid. The investigators reported that myoclonus, seizures, and pupillary light reflex dysfunction improved, leading to the recovery of gross motor function in two patients (Narita et al., 2016). Another study of the long-term safety and efficacy of combined high-dose ABX up to 21 mg/kg/day) and ERT in GD3 showed that during the first 2 years seizure frequency and neurocognitive function worsened, but after the ABX dosage was increased to 27 mg/kg/day, seizure frequency markedly decreased from the baseline, neurocognitive function improved and the drug was tolerated without severe adverse events (Kim et al., 2019). However, these are primarily antidotal reports, and a double-blind placebo-control study is needed. ABX is currently being tested under a single-center phase II clinical trial in 75 subjects with mild to moderate PD by randomizing participants into ABX high-dose (1,050 mg/day), low-dose (525 mg/day), or placebo group (Silveira et al., 2019), and reasonable cerebrospinal fluid levels were attained (Mullin et al., 2020).

## Non-Inhibitory Chaperones

A major limitation of inhibitory chaperones is that the chaperone activity must be balanced against the functional inhibition of GCase. In contrast, non-inhibitory chaperones can assist in the folding of mutant GCase in the ER and the translocation to lysosomes without interfering with the active site of the enzyme, and thus can restore enzyme activity in the lysosome. Several non-inhibitory compounds were identified by a high throughput screening (HTS) assay using an extract of spleen from a patient with GD as the source of mutant enzyme (Goldin et al., 2012). In this screen, saposin C and other potential cofactors were present in the extract, likely enhancing the detection of non-inhibitory chaperones. The activities of the compounds were confirmed in subsequent cell-based assays using patient-derived fibroblasts. The screen yielded novel pyrazolopyrimidine-based non-inhibitory pharmacological chaperones (Patnaik et al., 2012). Aflaki et al. (2014) assessed the efficacy of one non-inhibitory chaperone identified by HTS as a drug candidate for GD or *GBA1*-PD. Since fibroblasts do not store GlcCer, monocyte-derived macrophages from 20 patients with GD and GD iPSC-differentiated macrophages were examined. The non-inhibitory chaperone, NCGC758, enhanced GCase activity, reduced glycolipid storage, and normalized chemotaxis and the production of reactive oxygen species in the macrophages. NCGC758 also reversed inflammatory defects in GD macrophages by inducing autophagy and reducing IL-1b secretion (Aflaki et al., 2016b). Another lead non-inhibitory small molecule, NCGC607, was tested in dopaminergic (DA) neurons differentiated from type 1 and 2 GD and GD+PD iPSCs (Aflaki et al., 2016a). NCGC607 restored GCase activity and protein levels, and reduced glycolipid storage in GD DA neurons, indicating its therapeutic potential for GD. In addition, NCGC607 reduced α-Syn levels in DA neurons from patients with parkinsonism, suggesting that NCGC607 or a derivative of this lead compound may have efficacy as a treatment for PD (Aflaki et al., 2016a). NCGC758 also reduced α-Syn levels and improved lysosomal function in iPSC-derived DA neurons differentiated from patients with GD and PD (Mazzulli et al., 2016b).

## Other Small Molecules

Histone deacetylase inhibitors (HDACi) have also been explored for the treatment of GD by modulating a GCase-associated ubiquitin–proteasome pathway (Lu et al., 2011). It was shown that a known HDACi (SAHA) and a unique small-molecule HDACi (LB-205) rescued GCase levels and increased enzymatic activity in fibroblasts derived from patients with GD1 and GD2. It was further shown that HDACi inhibits the deacetylation of heat shock protein (HSP90), resulting in impaired recognition of the mutant peptide by HSP90, thus protecting GCase from degradation (Yang et al., 2013).

Recently, S-181, a new small-molecule capable of stabilizing wild-type GCase was developed. S-181 increased GCase activity in iPSC-derived DA neurons from patients with idiopathic PD, as well as in patients with PD carrying *GBA1* mutation c.84dupG and mutations in other PD genes (Burbulla et al., 2019). S-181 treatment in these DA neurons reversed pathogenic phenotypes including the reduced accumulation of oxidized dopamine. It was also shown in Table 1 that treating wild-type and heterozygous D409V mice with S-181 increased GCase activity in both, resulting in reduction of the lipid substrates and α-Syn in brain (Burbulla et al., 2019).

**TABLE 1:** Small molecules considered as therapeutics for GD and *GBA1*-associated PD.

**Name of small molecules**	**Disease modeled**	***GBA1* mutations tested**	**Model organisms**	**Effect of small molecules**	**References**
**Inhibitory chaperones**					
NN-DNJ	GD1	N370S	Fibroblasts	Increased GCase activity	Sawkar et al., 2002
α-1-C-Nonyl-DIX	GD1	N370S	Fibroblasts	Increased GCase activity	Compain et al., 2006
sp2-iminosugar	GD1	F213I, N370S	Fibroblasts	Increased GCase activity	Luan et al., 2009
	nGD	L444P, G202R	DA neurons from iPSCs	Increased GCase activity and protein level	Tiscornia et al., 2013
Isofagomine	GD1	N370S	Fibroblasts	Increased GCase activity	Lieberman et al., 2007
	nGD	L444P	Fibroblasts and lymphoblasts	Increased GCase activity Reduced GlcCer levels	Khanna et al., 2010
	nGD	L444P	Mouse	Increased GCase activity in relevant tissues	Khanna et al., 2010
	nGD	V394L/V394L + saposin C−/−	Mouse	Extended lifespan Increased GCase activity and protein levels Attenuation of proinflammatory response	Sun et al., 2011
	nGD	V394L, D409H, and D409V	Mouse	Increased GCase activity in relevant tissues	Sun et al., 2012
	PD	α-Syn overexpression	Mouse	Improved motor function Reduced α-Syn immunoreactivity Reduced α-Syn aggregates	Richter et al., 2014
	nGD	V394L/V394L + saposin C−/−	Mouse	Extended lifespan Attenuation of proinflammatory response Altered expression of DEGs*	Dasgupta et al., 2015
	*GBA1*-PD	N370S, L444P	Drosophila	Decreased ER** stress Restored motor function	Sanchez-Martinez et al., 2016
NAdBT-AIJ + coQ	nGD	L444P	Mouse	Restored mitochondrial dysfunction Increased GCase activity	De La Mata et al., 2015
α-1-C-tridecyl-DAB	GD1	N370S	Fibroblasts	Increased GCase activity	Kato et al., 2016
Ambroxol	GD1	N370S, F213I	Fibroblasts and lymphoblasts	Increased GCase activity and protein levels Reduced GlcCer levels	Maegawa et al., 2009
	GD1,2&3	N370S, F213I, N188S, G193W, R120W	Fibroblasts	Increased GCase activity	Luan et al., 2013
	GD1,2&3	N370S, V394L, R120W, R415R, R131C	Fibroblasts	Increased GCase activity	Bendikov Bar et al., 2013
	GD1 and *GBA1*-PD	N370S, etc.	Fibroblasts	Increased GCase activity and protein levels increase GCase mRNA and TFEB mRNA	McNeill et al., 2014
	*GBA1*-PD and non-*GBA1*-PD	N370S, L444P	Fibroblasts, drosophila	Increased GCase activity Increased Sap C	Ambrosi et al., 2015
	PD	L444P, α-Syn overexpression	Mouse	Increased GCase activity in brains Reduced α-Syn levels	Migdalska-Richards et al., 2016
	PD	wildtype	Non-human primate	Increased GCase activity in brains	Migdalska-Richards et al., 2017a
	nGD	C-terrminal 133aa deletion in *GBAb*	Drosophila	Reduced unfolded protein response Reduced neuroinflammation Enhanced lifespan	Cabasso et al., 2019
**Non-inhibitory Chaperonea**					
NCGC758	GD1 GD2	N370S, L444P, c.84dupG, IVS2+1	Macrophages from iPSCs	Increased GCase activity Reduced GlcCer levels Recovered ROS*** production Improved chemotaxis	Aflaki et al., 2014
NCGC758	GD1	N370S	Macrophages from iPSCs	Induced autophagy and Reduced IL-1β secretion	Aflaki et al., 2016a
NCGC758	nGD	N370S, c.84dupG	DA neurons from iPSCs	Increased GCase activity Reduced α-Syn levels Improved lysosomal function	Mazzulli et al., 2016b
NCGC607	GD, GD with parkinsonism	N370S, c.84dupG, IVS2+1, L444P	DA neurons from iPSCs	Increased GCase activity Reduced GlcCer and GlcSph levels Reduced α-Syn levels	Aflaki et al., 2016a
**Others**					
LB-250	GD	N370S, L444P	Fibroblasts	Inhibit histone deacetylase activity Increased GCase activity and protein levels	Lu et al., 2011
S-181	*GBA1*-PD and non-*GBA1*-PD	c.84dupG, wildtype	DA neurons from iPSCs	Increased GCase activity Reduced GlcCer levels Reduced the oxidized dopamine and α-Syn	Burbulla et al., 2019
S-181	*GBA1*-PD	D409V	Mouse	Reduced GlcCer and GlcSph levels in brain Reduced α-Syn levels in brain	Burbulla et al., 2019

*Differentially expressed brain mRNAs. **Endoplasmic reticulum. ***Reactive oxygen species.

## Discussion

There are several FDA-approved ERT (Cerezyme, etc.) and SRT (Zaversca, etc.) drugs to treat GD. However, ERT does not have utility for *GBA1*-PD since it does not cross the blood-brain- barrier and since substrate accumulation in *GBA1*-PD is minimal, it is unlikely that SRT will be efficacious (Sidransky et al., 2019). Small molecule pharmacological chaperones have been developed to overcome these limitations. The first iminosugar developed, NN-DNJ, increased GCase activity in cells from patients with GD (Sawkar et al., 2002) by strong binding to GCase (Thirumal Kumar et al., 2019). However, NN-DNJ only increases enzymatic activity in lines with specific *GBA1* mutations (Sawkar et al., 2002; Thirumal Kumar et al., 2019) and also inhibits GlcCer synthase activity in a dose-dependent manner (Compain et al., 2006). Isofagomine (IFG) appears to have more target-specific inhibition and broader pre-clinical efficacy in both *in vitro* and *in vivo* models of different types of GD and PD. However, like other inhibitory small molecule chaperones, IFG also causes dosage-dependent inhibition of GCase, limiting its clinical utility. A phase II clinical trial of IFG failed to improve clinical symptoms in patients with GD, likely due to dosing challenges. Ambroxol (ABX) has been evaluated as an inhibitory chaperone with fewer side effects and better efficacy, because its inhibitory effect does not occur at the low pH present in lysosomes. A recent comparative molecular docking analysis showed that ABX has more broad binding affinity toward GCase than NN-DNJ and other SRT drugs (Thirumal Kumar et al., 2019). A phase II clinical study of ABX for PD is currently underway (Silveira et al., 2019).

Non-inhibitory chaperones are highly attractive candidate drugs for the treatment of GD or *GBA1*-PD because they avoid the primary problem associated with inhibitory chaperones, the inhibitory competition with substrates of GCase in the lysosome. A few non-inhibitory chaperones identified by HTS were shown to have disease-reversing effects in cellular models of GD or *GBA1*-PD (Aflaki et al., 2014, 2016a). However, there are also difficulties in developing non-inhibitory chaperones. The binding of non-inhibitory chaperones to sites other than the active site makes it difficult to perform structure-guided optimization of drug efficacy and challenging to evaluate the potential activity of candidate molecules from HTS. To resolve this problem, visualizing the activity of endogenous levels of GCase in live cells is required. This might be achieved through the development of a fluorescence-based substrate probe representing GCase activity in lysosomes of live cells (Jung et al., 2016).

To increase the likelihood of success in clinical trials, a properly designed pre-clinical drug testing strategy is essential. Thus, appropriate *in vitro* and *in vivo* disease models which mimic key features of the disease are cessary. DA neurons differentiated from patient iPSCs accumulate α-Syn and oxidized dopamine, and thus can be used to test the efficacy of small molecules for PD (Aflaki et al., 2016a; Burbulla et al., 2019). However, mouse models accurately replicating PD phenotypes are still needed. A previous study showed that mice carrying the L444P mutation had increased stability of over-expressed human wild-type and A53T mutant α-Syn (Fishbein et al., 2014). However, the L444P mice did not demonstrate accumulation of α-Syn and there was no PD phenotypes in the L444P carrier mice (Migdalska-Richards et al., 2017b). While heterozygous L444P mice overexpressing mutant α-Syn had exacerbated motor deficits (Fishbein et al., 2014) and enhanced dopaminergic neurodegeneration (Migdalska-Richards et al., 2017b), it is not clear whether this phenotype can be used to establish drug efficacy, especially as in patients, the α-Syn levels are not as high, and the protein is not mutated. To identify and optimize candidate non-inhibitory chaperones, new methods for evaluating GCase activity in live cells and more appropriate *GBA1*-PD animal models need to be developed. Three dimensional *in-vitro* cell culture systems can be also considered for drug screening and preclinical validation of candidate drugs, because they more closely resembles the *in-vivo* cell environment than routine *in-vitro* cell culture (Langhans, 2018).

## Author Contributions

T-UH contributed to the conceptual idea, reviewed, analyzed the literature, and wrote the manuscript and table. RS assisted in compiling background material and in writing the manuscript. ES provided the conceptual idea, reviewed, and edited the manuscript.

## Conflict of Interest

The authors declare that the research was conducted in the absence of any commercial or financial relationships that could be construed as a potential conflict of interest.

## References

[B1] Aflaki E. Borger D. K. Moaven N. Stubblefield B. K. Rogers S. A. Patnaik S. (2016a). A new glucocerebrosidase chaperone reduces α-synuclein and glycolipid levels in iPSC-derived dopaminergic neurons from patients with gaucher disease and parkinsonism. *J. Neurosci.* 36 7441–7452. 10.1523/JNEUROSCI.0636-16.2016 27413154 PMC4945664

[B2] Aflaki E. Moaven N. Borger D. K. Lopez G. Westbroek W. Chae J. J. (2016b). Lysosomal storage and impaired autophagy lead to inflammasome activation in Gaucher macrophages. *Aging Cell* 15 77–88. 10.1111/acel.12409 26486234 PMC4717273

[B3] Aflaki E. Stubblefield B. K. Maniwang E. Lopez G. Moaven N. Goldin E. (2014). Macrophage models of Gaucher disease for evaluating disease pathogenesis and candidate drugs. *Sci. Transl. Med.* 6:240ra273. 10.1126/scitranslmed.3008659 24920659 PMC4161206

[B4] Ambrosi G. Ghezzi C. Zangaglia R. Levandis G. Pacchetti C. Blandini F. (2015). Ambroxol-induced rescue of defective glucocerebrosidase is associated with increased LIMP-2 and saposin C levels in GBA1 mutant Parkinson’s disease cells. *Neurobiol. Dis.* 82 235–242. 10.1016/j.nbd.2015.06.008 26094596

[B5] Balestrino R. Schapira A. H. V. (2018). Glucocerebrosidase and Parkinson disease: molecular, clinical, and therapeutic implications. *Neuroscientist* 24 540–559. 10.1177/1073858417748875 29400127

[B6] Bendikov Bar I. Maor G. Filocamo M. Horowitz M. (2013). Ambroxol as a pharmacological chaperone for mutant glucocerebrosidase. *Blood Cells Mol. Dis.* 50 141–145. 10.1016/j.bcmd.2012.10.007 23158495 PMC3547170

[B7] Bennett L. L. Turcotte K. (2015). Eliglustat tartrate for the treatment of adults with type 1 Gaucher disease. *Drug Des. Devel. Ther.* 9 4639–4647. 10.2147/DDDT.S77760 26345314 PMC4554398

[B8] Beutler E. Grabowski G. A. (2001). “Gaucher disease,” in *The Metabolic & Molecular Bases of Inherited Disease*, ed. Scriver C. R. (New York: McGraw-Hill), 3635–3668.

[B9] Burbulla L. F. Jeon S. Zheng J. Song P. Silverman R. B. Krainc D. (2019). A modulator of wild-type glucocerebrosidase improves pathogenic phenotypes in dopaminergic neuronal models of Parkinson’s disease. *Sci. Transl. Med.* 11:eaau6870. 10.1126/scitranslmed.aau6870 31619543 PMC7359409

[B10] Cabasso O. Paul S. Dorot O. Maor G. Krivoruk O. Pasmanik-Chor M. (2019). *Drosophila melanogaster* mutated in its GBA1b ortholog recapitulates neuronopathic gaucher disease. *J. Clin. Med.* 8:1420. 10.3390/jcm8091420 31505865 PMC6780790

[B11] Compain P. Martin O. R. Boucheron C. Godin G. Yu L. Ikeda K. (2006). Design and synthesis of highly potent and selective pharmacological chaperones for the treatment of Gaucher’s disease. *Chembiochem* 7 1356–1359. 10.1002/cbic.200600217 16871601

[B12] Dasgupta N. Xu Y. H. Li R. Peng Y. Pandey M. K. Tinch S. L. (2015). Neuronopathic Gaucher disease: dysregulated mRNAs and miRNAs in brain pathogenesis and effects of pharmacologic chaperone treatment in a mouse model. *Hum. Mol. Genet.* 24 7031–7048. 10.1093/hmg/ddv404 26420838 PMC4654057

[B13] De La Mata M. Cotan D. Oropesa-Avila M. Garrido-Maraver J. Cordero M. D. Villanueva Paz M. (2015). Pharmacological chaperones and coenzyme Q10 treatment improves mutant β-glucocerebrosidase activity and mitochondrial function in neuronopathic forms of gaucher disease. *Sci. Rep.* 5:10903. 10.1038/srep10903 26045184 PMC4456666

[B14] Fishbein I. Kuo Y. M. Giasson B. I. Nussbaum R. L. (2014). Augmentation of phenotype in a transgenic Parkinson mouse heterozygous for a Gaucher mutation. *Brain* 137 3235–3247. 10.1093/brain/awu291 25351739 PMC4240298

[B15] Gegg M. E. Burke D. Heales S. J. Cooper J. M. Hardy J. Wood N. W. (2012). Glucocerebrosidase deficiency in substantia nigra of parkinson disease brains. *Ann. Neurol.* 72 455–463. 10.1002/ana.23614 23034917 PMC3638323

[B16] Goldin E. Zheng W. Motabar O. Southall N. Choi J. H. Marugan J. (2012). High throughput screening for small molecule therapy for Gaucher disease using patient tissue as the source of mutant glucocerebrosidase. *PLoS One* 7:e29861. 10.1371/journal.pone.0029861 22272254 PMC3260169

[B17] Gupta N. Oppenheim I. M. Kauvar E. F. Tayebi N. Sidransky E. (2011). Type 2 Gaucher disease: phenotypic variation and genotypic heterogeneity. *Blood Cells Mol. Dis.* 46 75–84. 10.1016/j.bcmd.2010.08.012 20880730 PMC3018671

[B18] Henley W. E. Anderson L. J. Wyatt K. M. Nikolaou V. Anderson R. Logan S. (2014). The NCS-LSD cohort study: a description of the methods and analyses used to assess the long-term effectiveness of enzyme replacement therapy and substrate reduction therapy in patients with lysosomal storage disorders. *J. Inherit. Metab. Dis.* 37 939–944. 10.1007/s10545-014-9679-6 24519353

[B19] Horowitz M. Pasmanik-Chor M. Borochowitz Z. Falik-Zaccai T. Heldmann K. Carmi R. (1998). Prevalence of glucocerebrosidase mutations in the Israeli Ashkenazi Jewish population. *Hum. Mutat.* 12 240–244. 10.1002/(sici)1098-1004(1998)12:4<240::aid-humu4>3.0.co;2-j 9744474

[B20] Jung O. Patnaik S. Marugan J. Sidransky E. Westbroek W. (2016). Progress and potential of non-inhibitory small molecule chaperones for the treatment of Gaucher disease and its implications for Parkinson disease. *Expert Rev. Proteomics* 13 471–479. 10.1080/14789450.2016.1174583 27098312 PMC5381920

[B21] Kato A. Nakagome I. Sato K. Yamamoto A. Adachi I. Nash R. J. (2016). Docking study and biological evaluation of pyrrolidine-based iminosugars as pharmacological chaperones for Gaucher disease. *Org. Biomol. Chem.* 14 1039–1048. 10.1039/c5ob02223a 26633162

[B22] Khanna R. Benjamin E. R. Pellegrino L. Schilling A. Rigat B. A. Soska R. (2010). The pharmacological chaperone isofagomine increases the activity of the Gaucher disease L444P mutant form of β-glucosidase. *FEBS J.* 277 1618–1638. 10.1111/j.1742-4658.2010.07588.x 20148966 PMC2874831

[B23] Kim Y. M. Yum M. S. Heo S. H. Kim T. Jin H. K. Bae J. S. (2019). Pharmacologic properties of high-dose ambroxol in four patients with Gaucher disease and myoclonic epilepsy. *J. Med. Genet.* 57 124–131. 10.1136/jmedgenet-2019-106132 31649052 PMC7029246

[B24] Langhans S. A. (2018). Three-Dimensional *in vitro* cell culture models in drug discovery and drug repositioning. *Front. Pharmacol.* 9:6 10.3389/fphar.2018.00006 PMC5787088 29410625

[B25] Lieberman R. L. Wustman B. A. Huertas P. Powe A. C. Pine C. W. Khanna R. (2007). Structure of acid β-glucosidase with pharmacological chaperone provides insight into Gaucher disease. *Nat. Chem. Biol.* 3 101–107. 10.1038/nchembio850 17187079

[B26] Lu J. Yang C. Chen M. Ye D. Y. Lonser R. R. Brady R. O. (2011). Histone deacetylase inhibitors prevent the degradation and restore the activity of glucocerebrosidase in Gaucher disease. *Proc. Natl. Acad. Sci. U.S.A.* 108 21200–21205. 10.1073/pnas.1119181109 22160715 PMC3248545

[B27] Luan Z. Higaki K. Aguilar-Moncayo M. Li L. Ninomiya H. Nanba E. (2010). A fluorescent sp^2^-iminosugar with pharmacological chaperone activity for gaucher disease: synthesis and intracellular distribution studies. *Chembiochem* 11 2453–2464. 10.1002/cbic.201000323 21064079

[B28] Luan Z. Higaki K. Aguilar-Moncayo M. Ninomiya H. Ohno K. Garcia-Moreno M. I. (2009). Chaperone activity of bicyclic nojirimycin analogues for Gaucher mutations in comparison with N-(n-nonyl)deoxynojirimycin. *Chembiochem* 10 2780–2792. 10.1002/cbic.200900442 19830760

[B29] Luan Z. Li L. Higaki K. Nanba E. Suzuki Y. Ohno K. (2013). The chaperone activity and toxicity of ambroxol on Gaucher cells and normal mice. *Brain Dev.* 35 317–322. 10.1016/j.braindev.2012.05.008 22682976

[B30] Lukina E. Watman N. Dragosky M. Lau H. Avila Arreguin E. Rosenbaum H. (2019). Outcomes after 8 years of eliglustat therapy for Gaucher disease type 1: Final results from the Phase 2 trial. *Am. J. Hematol.* 94 29–38. 10.1002/ajh.25300 30264864 PMC6587500

[B31] Maegawa G. H. Tropak M. B. Buttner J. D. Rigat B. A. Fuller M. Pandit D. (2009). Identification and characterization of ambroxol as an enzyme enhancement agent for Gaucher disease. *J. Biol. Chem.* 284 23502–23516. 10.1074/jbc.M109.012393 19578116 PMC2749124

[B32] Mazzulli J. R. Zunke F. Isacson O. Studer L. Krainc D. (2016a). α-Synuclein-induced lysosomal dysfunction occurs through disruptions in protein trafficking in human midbrain synucleinopathy models. *Proc. Natl. Acad. Sci. U.S.A.* 113 1931–1936. 10.1073/pnas.1520335113 26839413 PMC4763774

[B33] Mazzulli J. R. Zunke F. Tsunemi T. Toker N. J. Jeon S. Burbulla L. F. (2016b). Activation of β-glucocerebrosidase reduces pathological α-synuclein and restores lysosomal function in Parkinson’s patient midbrain neurons. *J. Neurosci.* 36 7693–7706. 10.1523/JNEUROSCI.0628-16.2016 27445146 PMC4951575

[B34] McNeill A. Magalhaes J. Shen C. Chau K. Y. Hughes D. Mehta A. (2014). Ambroxol improves lysosomal biochemistry in glucocerebrosidase mutation-linked Parkinson disease cells. *Brain* 137 1481–1495. 10.1093/brain/awu020 24574503 PMC3999713

[B35] Mena-Barragan T. Garcia-Moreno M. I. Nanba E. Higaki K. Concia A. L. Clapes P. (2016). Inhibitor versus chaperone behaviour of D-fagomine, DAB and LAB sp(2)-iminosugar conjugates against glycosidases: a structure-activity relationship study in Gaucher fibroblasts. *Eur. J. Med. chem.* 121 880–891. 10.1016/j.ejmech.2015.08.038 26361824

[B36] Migdalska-Richards A. Daly L. Bezard E. Schapira A. H. (2016). Ambroxol effects in glucocerebrosidase and α-synuclein transgenic mice. *Ann. Neurol.* 80 766–775. 10.1002/ana.24790 27859541 PMC5132106

[B37] Migdalska-Richards A. Ko W. K. D. Li Q. Bezard E. Schapira A. H. V. (2017a). Oral ambroxol increases brain glucocerebrosidase activity in a nonhuman primate. *Synapse* 71:e21967. 10.1002/syn.21967 28295625 PMC5485051

[B38] Migdalska-Richards A. Wegrzynowicz M. Rusconi R. Deangeli G. Di Monte D. A. Spillantini M. G. (2017b). The L444P Gba1 mutation enhances α-synuclein induced loss of nigral dopaminergic neurons in mice. *Brain* 140 2706–2721. 10.1093/brain/awx221 28969384 PMC5841155

[B39] Mistry P. K. Balwani M. Baris H. N. Turkia H. B. Burrow T. A. Charrow J. (2018). Safety, efficacy, and authorization of eliglustat as a first-line therapy in Gaucher disease type 1. *Blood Cells Mol. Dis.* 71 71–74. 10.1016/j.bcmd.2018.04.001 29680197

[B40] Mullin S. Hughes D. Mehta A. Schapira A. H. V. (2019). Neurological effects of glucocerebrosidase gene mutations. *Eur. J. Neurol.* 26:e0388-29. 10.1111/ene.13837 30315684 PMC6492454

[B41] Mullin S. Smith L. Lee K. D’souza G. Woodgate P. Elflein J. (2020). Ambroxol for the treatment of patients with parkinson disease with and without glucocerebrosidase gene mutations: a nonrandomized, noncontrolled trial. *JAMA Neurol.* 13:e194611. 10.1001/jamaneurol.2019.4611 31930374 PMC6990847

[B42] Murphy K. E. Gysbers A. M. Abbott S. K. Tayebi N. Kim W. S. Sidransky E. (2014). Reduced glucocerebrosidase is associated with increased α-synuclein in sporadic Parkinson’s disease. *Brain* 137 834–848. 10.1093/brain/awt367 24477431 PMC3927701

[B43] Nalls M. A. Duran R. Lopez G. Kurzawa-Akanbi M. Mckeith I. G. Chinnery P. F. (2013). A multicenter study of glucocerebrosidase mutations in dementia with Lewy bodies. *JAMA Neurol.* 70 727–735. 10.1001/jamaneurol.2013.1925 23588557 PMC3841974

[B44] Narita A. Shirai K. Itamura S. Matsuda A. Ishihara A. Matsushita K. (2016). Ambroxol chaperone therapy for neuronopathic Gaucher disease: a pilot study. *Ann. Clin. Transl. Neurol.* 3 200–215. 10.1002/acn3.292 27042680 PMC4774255

[B45] Pastores G. M. Patel M. J. Firooznia H. (2000). Bone and joint complications related to Gaucher disease. *Curr. Rheumatol. Rep.* 2 175–180. 10.1007/s11926-000-0059-x 11123056

[B46] Patnaik S. Zheng W. Choi J. H. Motabar O. Southall N. Westbroek W. (2012). Discovery, structure-activity relationship, and biological evaluation of noninhibitory small molecule chaperones of glucocerebrosidase. *J. Med. Chem.* 55 5734–5748. 10.1021/jm300063b 22646221 PMC3400126

[B47] Pawlinski L. Malecki M. T. Kiec-Wilk B. (2018). The additive effect on the antiepileptic treatment of ambroxol in type 3 Gaucher patient. The early observation. *Blood Cells Mol. Dis.* 68 192–193. 10.1016/j.bcmd.2016.12.001 28007403

[B48] Poewe W. Seppi K. Tanner C. M. Halliday G. M. Brundin P. Volkmann J. (2017). Parkinson disease. *Nat. Rev. Dis. Primers* 3:17013. 10.1038/nrdp.2017.13 28332488

[B49] Richter F. Fleming S. M. Watson M. Lemesre V. Pellegrino L. Ranes B. (2014). A GCase chaperone improves motor function in a mouse model of synucleinopathy. *Neurotherapeutics* 11 840–856. 10.1007/s13311-014-0294-x 25037721 PMC4391384

[B50] Sala G. Marinig D. Arosio A. Ferrarese C. (2016). Role of chaperone-mediated autophagy dysfunctions in the pathogenesis of Parkinson’s disease. *Front. Mol. Neurosci.* 9:157. 10.3389/fnmol.2016.00157 28066181 PMC5179559

[B51] Sanchez-Martinez A. Beavan M. Gegg M. E. Chau K. Y. Whitworth A. J. Schapira A. H. (2016). Parkinson disease-linked GBA mutation effects reversed by molecular chaperones in human cell and fly models. *Sci. Rep.* 6:31380. 10.1038/srep31380 27539639 PMC4990939

[B52] Sawkar A. R. Cheng W. C. Beutler E. Wong C. H. Balch W. E. Kelly J. W. (2002). Chemical chaperones increase the cellular activity of N370S β -glucosidase: a therapeutic strategy for Gaucher disease. *Proc. Natl. Acad. Sci. U.S.A.* 99 15428–15433. 10.1073/pnas.192582899 12434014 PMC137733

[B53] Schiffmann R. Fitzgibbon E. J. Harris C. Devile C. Davies E. H. Abel L. (2008). Randomized, controlled trial of miglustat in Gaucher’s disease type 3. *Ann. Neurol.* 64 514–522. 10.1002/ana.21491 19067373 PMC2605167

[B54] Sidransky E. (2004). Gaucher disease: complexity in a “simple” disorder. *Mol. Genet. Metab.* 83 6–15. 10.1016/j.ymgme.2004.08.015 15464415

[B55] Sidransky E. (2012). Gaucher disease: insights from a rare Mendelian disorder. *Discov. Med.* 14 273–281. 23114583 PMC4141347

[B56] Sidransky E. Arkadir D. Bauer P. Dinur T. Lopez G. Rolfs A. (2019). Substrate reduction therapy for GBA1-associated Parkinsonism: are we betting on the wrong mouse? *Mov. Disord.* 35 228–230. 10.1002/mds.27903 31710399

[B57] Sidransky E. Lopez G. (2012). The link between the GBA gene and parkinsonism. *Lancet Neurol.* 11 986–998. 10.1016/S1474-4422(12)70190-4 23079555 PMC4141416

[B58] Sidransky E. Nalls M. A. Aasly J. O. Aharon-Peretz J. Annesi G. Barbosa E. R. (2009). Multicenter analysis of glucocerebrosidase mutations in Parkinson’s disease. *N. Engl. J. Med.* 361 1651–1661. 10.1056/NEJMoa0901281 19846850 PMC2856322

[B59] Silveira C. R. A. Mackinley J. Coleman K. Li Z. Finger E. Bartha R. (2019). Ambroxol as a novel disease-modifying treatment for Parkinson’s disease dementia: protocol for a single-centre, randomized, double-blind, placebo-controlled trial. *BMC Neurol.* 19:20. 10.1186/s12883-019-1252-3 30738426 PMC6368728

[B60] Spillantini M. G. Schmidt M. L. Lee V. M. Trojanowski J. Q. Jakes R. Goedert M. (1997). α-synuclein in Lewy bodies. *Nature* 388 839–840. 10.1038/42166 9278044

[B61] Stojkovska I. Krainc D. Mazzulli J. R. (2018). Molecular mechanisms of α-synuclein and GBA1 in Parkinson’s disease. *Cell Tissue Res.* 373 51–60. 10.1007/s00441-017-2704-y 29064079 PMC5916529

[B62] Sun Y. Liou B. Xu Y. H. Quinn B. Zhang W. Hamler R. (2012). Ex vivo and in vivo effects of isofagomine on acid β-glucosidase variants and substrate levels in Gaucher disease. *J. Biol. Chem.* 287 4275–4287. 10.1074/jbc.M111.280016 22167193 PMC3281716

[B63] Sun Y. Ran H. Liou B. Quinn B. Zamzow M. Zhang W. (2011). Isofagomine in vivo effects in a neuronopathic Gaucher disease mouse. *PLoS One* 6:e19037. 10.1371/journal.pone.0019037 21533102 PMC3080394

[B64] Surmeier D. J. Obeso J. A. Halliday G. M. (2017). Parkinson’s disease is not simply a prion disorder. *J. Neurosci.* 37 9799–9807. 10.1523/JNEUROSCI.1787-16.2017 29021297 PMC5637112

[B65] Taguchi Y. V. Liu J. Ruan J. Pacheco J. Zhang X. Abbasi J. (2017). Glucosylsphingosine promotes α-synuclein pathology in mutant GBA-associated Parkinson’s disease. *J. Neurosci.* 37 9617–9631. 10.1523/JNEUROSCI.1525-17.2017 28847804 PMC5628407

[B66] Thirumal Kumar D. Iyer S. Christy J. P. Siva R. Tayubi I. A. George P. (2019). A comparative computational approach toward pharmacological chaperones (NN-DNJ and ambroxol) on N370S and L444P mutations causing Gaucher’s disease. *Adv. Protein Chem. Struct. Biol.* 114 315–339. 10.1016/bs.apcsb.2018.10.002 30635084

[B67] Tiscornia G. Vivas E. L. Matalonga L. Berniakovich I. Barragan Monasterio M. Eguizabal C. (2013). Neuronopathic Gaucher’s disease: induced pluripotent stem cells for disease modelling and testing chaperone activity of small compounds. *Hum. Mol. Genet.* 22 633–645. 10.1093/hmg/dds471 23118351

[B68] Valayannopoulos V. (2013). Enzyme replacement therapy and substrate reduction therapy in lysosomal storage disorders with neurological expression. *Handb. Clin. Neurol.* 113 1851–1857. 10.1016/B978-0-444-59565-2.00055-1 23622408

[B69] Weiss K. Gonzalez A. Lopez G. Pedoeim L. Groden C. Sidransky E. (2015). The clinical management of Type 2 Gaucher disease. *Mol. Genet. Metab.* 114 110–122. 10.1016/j.ymgme.2014.11.008 25435509 PMC4312716

[B70] Yang C. Rahimpour S. Lu J. Pacak K. Ikejiri B. Brady R. O. (2013). Histone deacetylase inhibitors increase glucocerebrosidase activity in Gaucher disease by modulation of molecular chaperones. *Proc. Natl. Acad. Sci. U.S.A.* 110 966–971. 10.1073/pnas.1221046110 23277556 PMC3549125

[B71] Yap T. L. Gruschus J. M. Velayati A. Westbroek W. Goldin E. Moaven N. (2011). α-synuclein interacts with Glucocerebrosidase providing a molecular link between Parkinson and Gaucher diseases. *J. Biol. Chem.* 286 28080–28088. 10.1074/jbc.M111.237859 21653695 PMC3151053

[B72] Zimran A. Altarescu G. Elstein D. (2013). Pilot study using ambroxol as a pharmacological chaperone in type 1 Gaucher disease. *Blood Cells Mol. Dis.* 50 134–137. 10.1016/j.bcmd.2012.09.006 23085429

[B73] Zimran A. Goldblatt J. Szer J. (2018). Should eliglustat be first line therapy for patients with type 1 Gaucher disease? Definitions of safety and efficacy. *Blood Cells Mol. Dis.* 68 14–16. 10.1016/j.bcmd.2017.09.003 28935503

